# Hospitalizations for mental and behavioral disorders due to alcohol and other psychoactive substance use among adolescents in Brazil, 2017-2022

**DOI:** 10.1590/S2237-96222024V33E20231110.EN

**Published:** 2024-07-08

**Authors:** Maria Theresa Leal Galvão, Maria Vitória de Deus Ramos Santos, Luciana Mesquita Brito, Thalia Alves de Oliveira Evaristo, Eduardo Lima de Sousa, Joaquim Neto Alencar Cunha Leitão, André Sousa Rocha

**Affiliations:** 1 Universidade Federal do Piauí, Centro de Ciências da Saúde, Teresina, PI, Brazil; 2 Centro Universitário UniNovafapi, Departamento de Medicina, Teresina, PI, Brazil; 3 Centro Universitário Inta, Departamento de Psicologia, Itapipoca, CE, Brazil

**Keywords:** Substance Use Disorders, Hospitalization, Time-Series Studies, Adolescent, Mental Disorders, Trastornos Relacionados con Sustancias, Hospitalización, Estudios de Series Temporales, Adolescente, Trastornos Mentales

## Abstract

**Objective:**

To assess the epidemiological profile and trend in hospitalizations for mental and behavioral disorders due to alcohol and other psychoactive substance use among Brazilian adolescents, between 2017 and 2022.

**Methods:**

This was a time-series study using data from the Hospital Information System of the Brazilian National Health System; the trend analysis was performed by estimating the annual percentage change (APC) of hospitalization rates per 100,000 inhabitants and respective confidence intervals (95%CI), using the Prais-Winsten method.

**Results:**

A total of 29,991 hospitalizations were recorded in the study period, with a decreasing trend observed, from 16.18/100,000 inhabitants in 2017 to 13.72/100,000 inhab. in 2022 (percent change of -2.65%; 95%CI -4.47;-0.80), a greater decline was found in males (-3.48%; 95%CI -5.20;-1.72), in the age group of 15 to 19 years (-2.79%; 95%CI -4.49;-1.06), in the South (-3.29%; 95%CI -5.37;-1.16) and Midwest (-3.64%; 95%CI -5.75;-1.49) regions of the country.

**Conclusion:**

Hospitalizations showed a decreasing trend in the study period, with sociodemographic disparities.

## Study contributions

**Main results:**

There were 29,991 hospitalizations for mental and behavioral disorders due to the use of alcohol and other drugs among adolescents in Brazil, in 2017-2022. The trend in hospitalization rates was decreasing in the South and Midwest regions and among males.

**Implications for services:**

The results showed a decreasing trend in hospitalizations for alcohol and other drug use among adolescents, enabling the planning of interventions and specific public health policies to address this issue.

**Perspectives:**

It is expected that the study results will contribute to more effective strategies to combat and prevent the use of psychoactive substances by adolescents.

## Resumen

**Objetivo:**

Analizar el perfil epidemiológico y la tendencia de las hospitalizaciones por trastornos mentales y de comportamiento relacionados con el uso de alcohol y otras sustancias psicoactivas en adolescentes brasileños, desde 2017 hasta 2022.

**Métodos:**

Estudio de serie temporal utilizando el Sistema de Información Hospitalaria del Sistema Único de Salud; se evaluó la tendencia mediante la estimación del cambio porcentual anual (CPA) de las tasas de hospitalización por cada 100,000 habitantes y sus respectivos intervalos mediante el método de Prais-Winsten.

**Resultados:**

Se registraron 29.991 hospitalizaciones durante el período y se observó una tendencia decreciente en Brasil, desde 16,18 hospitalizaciones por cada 100.000 habitantes en 2017 hasta 13,72 en 2022 (variación de -2,65%; IC₉₅_%_ -4,47;-0,80), con mayor declive en hombres (-3,48%; IC₉₅_%_ -5,20;-1,72), en la edad de 15 a 19 años (-2,79%; IC₉₅_%_ -4,49;-1,06), en las regiones del Sur (-3,29%; IC₉₅_%_ -5,37; -1,16) y Centro-Oeste (-3,64%; IC₉₅_%_ -5,75;-1,49).

**Conclusión:**

Las hospitalizaciones mostraron una tendencia decreciente en el período, con disparidades sociodemográficas.

## INTRODUCTION

Adolescence is the phase of life between childhood and adulthood, characterized by a complex process of growth and adaptation to new physical, psychological and environmental structures.^1^ Throughout this process socioeconomic factors, such as low family income, biological and genetic factors related to neurological development, as well as family dysfunction and certain environmental exposures, such as substance abuse and violent situations, contribute to creating an environment conducive to the onset of mental disorders in this population.^2^ ^),(^ ^3^ According to data from the World Health Organization, one in seven young people aged 10 to 19 years experiences a mental disorder.^4^

The Statute of the Child and Adolescent (*Estatuto da Criança e do Adolescente*), a law that provides for the comprehensive protection of children and adolescents, includes mental health care as one of its basic guidelines, including those related to the use of alcohol and other psychoactive substances.^5^ Despite this normative support, the II Brazilian Report on Drugs highlighted the increasingly early onset of substance use, which corroborates the occurrence of associated mental and behavioral disorders, in addition to possible acute conditions requiring hospital care.^6^

The national survey on drug use, conducted in 2017, included 138 demographic strata, revealing that approximately 7 million people (34.3%) aged under 18 years reported alcohol consumption at least once in their lifetime, with 22.2% reporting use within the past 12 months.^7^ According to the same survey, around 1.3 million adolescents aged 12 to 17 years had already consumed manufactured cigarettes in their lifetime, and for approximately 15 million individuals (12 to 65 years old) who reported having used some illicit substance during their lifetime, the average age at first use was 16.6 years.^7^

In this context, the earlier the experimentation with these substances, the greater the risks of short, medium, or long-term, direct or indirect harm, with potential consequences such as: physical health damage to different organs, family conflicts, impaired development, difficulties in school and interpersonal relationships, in addition to the risk of involvement in crime and violence resulting from exposure to different environments. without supervision of a responsible adult.^8^

The use of psychoactive substances and their implications for adolescents have been the subject of several studies in Brazil.^9^ ^),(^ ^10^ ^),(^ ^11^ ^)^ Research on hospitalizations for mental disorders due to the use of psychoactive substances among specific population, encompassing comprehensive nationwide data across the five national macro-regions are scarce. Conducting studies with such age-specific and geographic approaches, along with their characteristics, is important for the development of more integrated and effective mental health policies.

Given the importance of the topic and the scarcity of studies with this focus, this study aimed to analyze the epidemiological profile and trend in hospitalizations for mental and behavioral disorders due to alcohol and other psychoactive substance use among Brazilian adolescents, between 2017 and 2022.

## METHODS

*Study design*

This was a time-series study assessing hospitalizations for mental and behavioral disorders due to alcohol and other psychoactive substance use among adolescents in Brazil, from 2017 to 2022, using the number of hospitalizations recorded by place of hospitalization as the unit of analysis.

*Setting*

According to the latest census data released by the Brazilian Institute of Geography and Statistics (Instituto Brasileiro de Geografia e Estatística - IBGE), in 2022, Brazil had 203,062,512 inhabitants, of whom 28,050,903 were aged 10 to 19 years. The country is comprised of 5,570 municipalities, spread across 27 Federative Units and five national macro-regions, in the following decreasing order of population: Southeast region (41.8%), Northeast region (26.9%), South region (14.7%), North region (8.5%) and Midwest region (8.0%).^12^ The National Health Establishment Registry (Cadastro Nacional de Estabelecimentos Saúde - CNES), in 2023, recorded 32,097 beds dedicated to psychiatry and mental health, with 17,280 of them available for hospitalization via the Brazilian National Health System.^13^

Data on hospitalizations were obtained from the Hospital Information System of the Brazilian National Health System (*Sistema de Informações Hospitalares do Sistema Único de Saúde* - SIH/SUS), managed by the Ministry of Health, through its Specialized Health Care Secretariat, together with the State and Municipal Departments of Health. These data were extracted from the Brazilian National Health System Information Technology Department (*Departamento de Informática do SUS* - DATASUS) website, through an online consultation carried out in June 2023.^14^

*Participants*

Hospitalization records with diagnoses identified by codes F10 (Mental and behavioral disorders due to alcohol use) and F19 (Mental and behavioral disorders due to use of other psychoactive substances) were selected, as listed in Chapter V of the International Statistical Classification of Diseases and Related Health Problems 10^th^ Revision (ICD-10).^15^

*Variables*

The variables used for the analysis of hospitalizations for mental and behavioral disorders due to alcohol and other psychoactive substances use among adolescents were: year when hospital care took place (between 2017 and 2022); macro-region of residence (North; Northeast; Southeast; South; Midwest); sex (male; female); race/skin color (White, Black, mixed-race, Asian, Indigenous); age group (in years: 10 to 14; 15 to 19); average length of hospital stay (in days); and type of care (elective; emergency).

*Data source/measurement*

Data from SIH/SUS and population projections for adolescents (10 to 19 years old) in Brazil, provided by the IBGE for the period from 2017 to 2022, were used.^14^

*Data analysis*

In order to calculate the hospitalization rate, the number of hospitalizations for mental and behavioral disorders due to alcohol and other psychoactive substances use among adolescents, provided by the SUS, was used as the numerator, while the population projection for adolescents living in the same location and period considered, provided by the IBGE, multiplied by 100,000, was used as the denominator.

Temporal trend was analyzed using the Prais-Winsten generalized linear regression method. According to this method, the years of the time series from 2017 to 2022 were considered as independent variables (X), while the logarithms of hospitalization rates by region of residence, sex, and age group (10 to 14 and 15 to 19 years) were considered as dependent variables (Y). The base-10 logarithmic transformation applied to the dependent variables (Y) aims to reduce the heterogeneity of residual variance in the linear regression analysis, helping in trend identification. This regression model is appropriate for correcting serial autocorrelation in time series, related to the dependence of a measure over time, with its own values at previous moments. Serial autocorrelation was assessed using the Durbin-Watson test.^16^

The trend was estimated by calculating the annual percentage change (APC) = [-1 + 10b1] * 100%.The corresponding 95% confidence interval was obtained by the following formula = [-1 + 10b1mín.] * 100% até [-1 + 10b1máx.] * 100%. The trend was interpreted as follows: increasing, when the rate of change was positive; decreasing, when the rate of change was negative; and stable, when there was no significant difference. Results with a p-value <0.05 were considered significant.^16^

Data analysis was performed using the R software, version 4.2.2, with a 95% confidence level.

*Ethical aspects*

As the study used secondary data, provided by DATASUS, ethical review by the Research Ethics Committee was not required, in accordance with the Resolution No. 466 of the National Health Council, December 12, 2012.^17^

## RESULTS

Between 2017 and 2022, there were 29,991 hospitalizations for mental and behavioral disorders due to alcohol and other drug use among adolescents in Brazil. It could be seen that 72.1% (n = 21,624) of the hospitalizations occurred among males and 86.6% (n = 25,960) were adolescents aged 15 to 19 years. Regarding race/skin color, White was the most frequent, accounting for 44.9% (n = 13,467) of hospitalizations during the period (Table 1).

**Table 1:** Characteristics of hospitalizations for mental and behavioral disorders due to alcohol and other psychoactive substance use among adolescents (n = 29,991), recorded in the Hospital Information System of the Brazilian National Health System, by sex, age and race/skin color, Brazil, 2017-2022

Variables	2017	2018	2019	2020	2021	2022	Total	%
Sex								
Male	4,120	4,326	4,190	3,046	2,956	2,986	21,624	72.1
Female	1,315	1,513	1,670	1,224	1,281	1,364	8,367	27.9
Age group (years)								
10-14	679	820	815	586	542	589	4,031	13.4
15-19	4,756	5,019	5,045	3,684	3,695	3,761	25,960	86.6
Race/skin color								
White	2,337	2,772	2,665	1,985	1,732	1,976	13,467	44.9
Black	283	284	354	222	214	275	1,632	5.4
Mixed-race	1,436	1,569	1,694	1,279	1,370	1,444	8,792	29.3
Asian	61	64	65	47	60	40	337	1.1
Indigenous	7	8	10	9	4	2	40	0.1
No information provided	1,311	1,142	1,072	728	857	613	5,723	19.1

**Figure 1:**
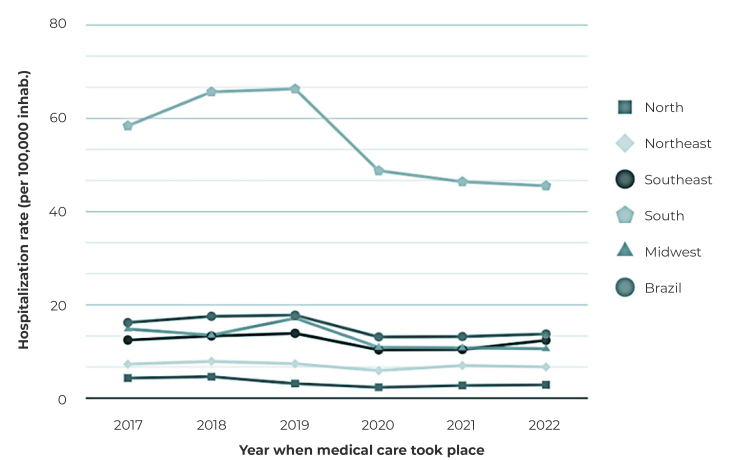
Distribution of hospitalization rates for mental and behavioral disorders due to alcohol and other psychoactive substance use among adolescents, by region of the country, Brazil, 2017-2022

Hospitalization rates for Brazil showed a reduction from 16.18/100,000 inhabitants. to 13.72/100,000 inhab. and a decreasing trend between 2017 and 2022 (Figure 1 and Table 2).

**Table 2:** Annual rates and trend of hospitalizations^a^ for mental and behavioral disorders due to alcohol and other psychoactive substance use among adolescents, by region of the country, sex and age group, Brazil, 2017-2022

Variables	2017	2018	2019	2020	2021	2022	Coefficient	p-value​	Change (%)	95%CI^c^	Trend^d^
Region											
North	​​4.27​	​​4.55​	​​3.08​	​​2.29​	​​2.68​	​​2.82​	​​-0.05	​​0.080​	​​-4.7	​​-8.42;-0.77	​​ Stable
​​Northeast​	​​7.22​	​​7.86​	​​7.34​	​​5.86​	​​6.96​	​​6.65​	​​-0.01	​​0.152​	​​-1.4​	​​-2.92;0.16​	​​Stable​
​​Southeast	​​12.43​	​​13.29​	​​13.88​	​​10.30​	​​10.38​	​​12.38​	​​-0.02	​​0.212​	​​-1.6​	​​-3.74;0.53​	​​Stable​
​​South	​​58.43​	​​65.70​	​​66.34​	​​48.77​	​​46.42​	​​45.55​	​​-0.03	​​0.039​	​​-3.3​	​​-5.37;-1.16	​​Decreasing​
​Midwest​	​​14.79​	​​13.48​	​​17.12​	​​10.86​	​​10.76​	​​10.57​	​​-0.04	​​0.030​	​​-3.6​	​​-5.75;-1.49	​​Decreasing​
Brazil	​​16.18​	​​17.53​	​​17.77​	​​13.10​	​​13.18​	​​13.72​	​​-0.03	​​0.049​	​​-2.7​	​​-4.47;-0.80	​​Decreasing​
Sex											
​​Male​	​​24.07​	​​25.48	​​24.92​	​​18.32​	​​18.02​	​​18.45​	​​-0.03	​​0.018	​​-3.5​	​​-5.20;-1.72	​​Decreasing​
​​Female​	​​7.98​	​​9.26​	​​10.33​	​​7.66​	​​8.13​	​​8.78​	​​-0.01	​​0.683​	​​-0.5​	​​-2.65;1.72​	​​Stable
Age group (years)											
​​10-14​	​​4.13​	​​5.06​	​​5.10​	​​3.72​	​​3.49​	​​3.85​	​​-0.02	​​0.163	​​-2.4​	​​-5.02;0.36​	​​Stable​
​​15-19	​​27,73​	​​29,37​	​​29,70​	​​21,87​	​​22,23​	​​22,93​	​​-0.03	​​0.035​	​​-2.8​	​​-4.49;-1.06	​​Decreasing​​

a) 95%CI = 95% confidence interval; b) Decreasing trend, when p-value < 0.05 and negative regression coefficient.

The highest hospitalization rates were observed in the South region, ranging from 58.43/100,000 inhab. to 45.55/100,000 inhabitants during the study period. The South (-3.3%; 95%CI -5.37;-1.16) and the Midwest (-3.6%; 95%CI -5.75;-1.49) regions showed decreasing trends in hospitalization rates, while in the other regions, they remained stable (Table 2).

**Figure 2:**
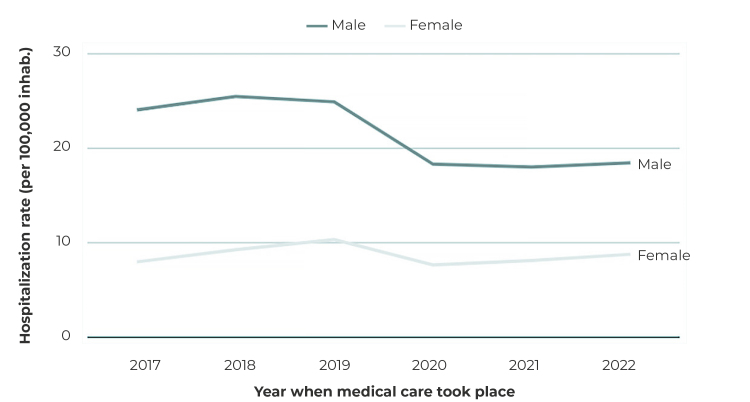
Distribution of hospitalization rates for mental and behavioral disorders due to alcohol and other psychoactive substance use among adolescents, by region of the country, Brazil, 2017-2022

Hospitalization rates in males showed a decreasing trend (-3.5%; 95%CI -5.20;-1.72), ranging from 24.07/100,000 inhab. to 18.45/100,000 inhab., while rates in females remained stable (Table 2 and Figure 2). As for the age groups analyzed, a decreasing trend in hospitalization rates was observed in the age group of 15 to 19 years (-2,8%; 95%CI -4.49;-1.06), ranging from 27.73/100,000 inhab. to 22.93/100,000 inhab. (Table 2).

The average length of hospital stay, related to the hospital admission authorizations approved during the analyzed period, was 16.4 days in 2017 and 15.8 days in 2022. The Northeast region showed the highest average (19.4 days), followed by the South (18.5 days), Southeast (14.4 days), Midwest (8.5 days) and North (6.2 days) regions. Regarding care, the majority of hospitalizations occurred as emergencies, accounting for 88.3% (26,483) of the total.

## DISCUSSION

In Brazil, from 2017 to 2022, hospitalization rates for mental and behavioral disorders due to alcohol and other psychoactive substance use among adolescents showed a decreasing trend, with a greater decline in males, in the age group of 15-19 years, and in the South and Midwest regions. It is noteworthy that the other variables used for the analysis of hospitalization rates showed a stable trend.

In the analysis of the selected period, the South region had a more significant number of hospitalizations. According to the *Study of Cardiovascular Risks in Adolescents* (ERICA), which took into consideration students aged 12 to 17 years, alcohol consumption was also higher in the South region, providing substantial support to the data found by this study.^18^ In addition, a time series analysis conducted in Paraná revealed that the main cause of hospitalizations in adolescents was mental and behavioral disorders caused by the use of psychoactive substances, accounting for 64.35% of hospitalizations in this age group between 2012 and 2015.^10^ One possible explanation for this phenomenon is that the perception of psychoactive substances use differs according to region. According to a study on adolescent alcohol-related behaviors, the Northern region of the country is associated with more reports of fear and insecurity regarding chemical dependence. In the South, a region where the consumption of wine is appreciated and celebrated, the influence of German and Italian colonial traditions on understanding alcohol use as less harmful to health is evident.^19^

Males accounted for the majority of hospitalizations for mental and behavioral disorders due to alcohol use among young Brazilians. An investigation on the same topic, conducted between 2010 and 2020, also found a higher rate of hospitalizations among young males, corroborating the findings of this study.^20^ This disparity between sexes can be attributed to several factors, such as (i) the national culture, marked by a persistent labeling of gender roles, and (ii) social and media environments, both influencing access to and acceptability of substance use among males.^21^ Similarly, according to the results found in the *II Brazilian Report on Drugs*, men showed higher hospitalization rates in all age groups, defining a consistent pattern observed in all Brazilian states.^6^ This reference in the literature suggests that there were no significant changes in the distribution of hospitalizations for psychoactive substance use, according to sex, during the study period.

The analysis of the results by age group highlights the highest hospitalization rates in the age group of 15 to 19 years. This fact is possibly related to the legality of alcohol in the country, as despite the legal restrictions on its consumption by minors, there is widespread access to the substance and it is difficult to monitor. There may also be a correlation between this finding and the significant influence of advertising campaigns promoting alcohol use, in addition to social pressure exerted by groups that encourage substance use.^11^ Family, educational or social contexts, in which each age group is situated, play a significant role in alcohol consumption and hospitalizations. Young people, aged 18 years and older, have free access to bars and nightclubs, which can increase alcohol consumption. These young people are also exposed to university environments, where peers often encourage the use of alcohol and psychoactive substances.^22^

Furthermore, the time of onset of chemical dependency varies depending on the influence of genetic, environmental, psychosocial and neurobiological factors. The drug exposure during adolescence may increase vulnerability to the development of drug addiction, due to the significant brain changes that occur in this age group, such as the development of reward and emotion regulation circuits and synaptic plasticity. This, once altered, can cause long-lasting adaptations in brain circuits, increasing the risk of addiction. Thus, the impact of age on the initiation and maintenance of the use of psychoactive substances is reinforced.^23^

The reduction in hospitalization rates between 2017 and 2022 can be attributed to several factors, such as restricted access to health services and the implementation of social distancing policies during the COVID-19 pandemic, when, according to the WHO, approximately 35% of mental health-related emergency services were interrupted.^24^At a university hospital in Rio Grande do Sul state, from October 2019 to October 2020, there was a reduction in the number of beds, from 30 to 20, for 60 days, as a preventive measure.^25^Another study, conducted in the United States in 2020, found an overall reduction in the use of psychoactive substances by adolescents during the pandemic.^26^ These findings demonstrate the impact of the COVID-19 emergency on hospitalizations for mental and behavioral disorders, which showed a decline in 2020 when compared to 2019, followed by an increase in 2021.^27^

The majority of the hospitalizations for mental and behavioral disorders analyzed here occurred as an emergency, a result supported by a study conducted in the North region, between 2017 and 2021, when 97% of hospitalizations were emergencies.^27^ The acute intoxication is described as acute intoxication as the most common clinical presentation of substance abuse among adolescents, manifested by psychomotor agitation, aggression, acute psychosis and, in the most severe cases, confusion and coma.²⁸ However, it is not possible to determine whether the hospitalizations occurred due to intoxication itself or secondary complications, given that the notification form does not provide space for this distinction.

A study involving individuals under 18 years of age, substance users, treated at a psychiatric emergency service in Marília, state of São Paulo, between 2000 and 2011, highlighted an increase in the frequency of using these services, related to (i) the growing prevalence of mental health problems in this age group, (ii) difficulty accessing community health services, and (iii) the stigma associated with mental disorders in other health facilities.^29^ Psychiatric emergency services are usually the first and sometimes the only source of care for child and adolescent health, emphasizing the importance of recognizing these services for (i) the development of effective intervention protocols, with clear guidelines for action, referral and return, and (ii) the development of procedures capable of encompassing the psychosocial factors underlying this demand.

The present study has some limitations inherent to its design. The aggregated information by it provides aggregate information by geographic region or location of hospitalization, which may obscure individual nuances and confounding related to incorrect or incomplete filling in of information in hospital records may also be present. Coding errors in diagnoses and procedures, as well as lack of detail regarding individuals’ clinical conditions, may compromise the quality of the data analyzed, making an accurate assessment of the results difficult.

Another limitation of this research is related to socioeconomic issues. It is possible that the diversity of the population in need of medical care has not been fully captured. The exclusion of those who did not seek hospital care, or who opted for private medical care, introduces an underestimation bias in the true prevalence of mental and behavioral disorders due to psychoactive substance use.

It is essential to recognize these limitations when interpreting the results of this study. Therefore, it is recommended to consider complementary approaches, such as cohort studies or qualitative research, in order to obtain a more comprehensive and accurate understanding of these public health issues, taking into account differences, or even socioeconomic disparities, in the general population.

During the period analyzed, there was a decreasing trend in hospitalizations for mental and behavioral disorders due to psychoactive substance use among Brazilian adolescents, with disparities between the national macro-regions and a higher prevalence of hospitalizations among young males, White race/skin color and age group of 15 to 19 years. Given these results, it is crucial to emphasize the importance of this study and the information it provides, essential for planning and targeting public policies and mental health practices aimed at the Brazilian adolescent population. There is a need for further research to deepen the discussion and provide new contributions to the topic, such as longitudinal studies or controlled interventions, as well as research exploring individual-level information, such as socioeconomic factors, family history, and access to prevention and intervention programs, enabling the identification of groups at greater vulnerability and guiding the development of better targeted interventions.
