# Supplementary material for: Hospitalizations for mental and behavioral disorders due to alcohol and other psychoactive substance use among adolescents in Brazil, 2017-2022
Source: Epidemiol Serv Saude. 2024 Jul 8;33:e20231110. doi: 10.1590/S2237-96222024V33E20231110.EN (PMC11251450; doi:10.1590/S2237-96222024V33E20231110.EN)
Supplement: Translation

# Translation: Hospitalizações por transtornos mentais e comportamentais relacionados ao uso de álcool e outras substâncias psicoativas em adolescentes no Brasil, 2017-2022

**DOI:** 10.1590/S2237-96222024V33E20231110.PT

**Authors:** Maria Theresa Leal Galvão, Maria Vitória de Deus Ramos Santos, Luciana Mesquita Brito, Thalia Alves de Oliveira Evaristo, Eduardo Lima de Sousa, Joaquim Neto Alencar Cunha Leitão, André Sousa Rocha

**Affiliations:** 1 Universidade Federal do Piauí, Centro de Ciências da Saúde, Teresina, PI, Brasil; 2 Centro Universitário UniNovafapi, Departamento de Medicina, Teresina, PI, Brasil; 3 Centro Universitário Inta, Departamento de Psicologia, Itapipoca, CE, Brasil

**Keywords:** Transtornos Relacionados ao Uso de Substâncias, Hospitalização, Estudos de Séries Temporais, Adolescente, Transtornos Mentais

## Abstract

**Objetivo:**

Analisar o perfil epidemiológico e a tendência das internações hospitalares por transtornos mentais e comportamentais devido ao uso de álcool e de outras substâncias psicoativas em adolescentes brasileiros, de 2017 a 2022.

**Métodos:**

Estudo de série temporal com dados do Sistema de Informações Hospitalares do Sistema Único de Saúde. A tendência foi analisada estimando-se a variação percentual anual (VPA) das taxas de internação por 100 mil habitantes e respectivos Intervalos pelo método de Prais-Winsten.

**Resultados:**

Foram registradas 29.991 internações no período, sendo observada uma tendência decrescente das internações no Brasil, variando de 16,18 internações por 100 mil hab., em 2017, para 13,72 em 2022 (variação de -2,65%; IC₉₅_%_ -4,47; -0,80), com maior declínio no sexo masculino (-3,48%; IC₉₅_%_ -5,20;-1,72), na faixa etária de 15 a 19 anos (-2,79%; IC₉₅_%_ -4,49;-1,06), nas regiões Sul (-3,29%; IC₉₅_%_ -5,37;-1,16) e Centro-Oeste (-3,64%; IC₉₅_%_ -5,75;-1,49).

**Conclusão:**

As internações apresentaram tendência decrescente no período, com disparidades sociodemográficas.

## Contribuições do estudo

**Principais resultados:**

Ocorreram 29.991 internações por transtornos mentais e comportamentais devido ao uso de álcool e outras drogas em adolescentes no Brasil, entre 2017 e 2022. A tendência das taxas de internações foi decrescente no Sul e Centro-Oeste e no sexo masculino.

**Implicações para os serviços:**

Os resultados apontaram tendência decrescente nas hospitalizações por uso de álcool e outras drogas entre os adolescentes, o que permite o planejamento de intervenções e de políticas públicas de saúde específicas para lidar com essa questão.

**Perspectivas:**

Espera-se que os resultados do estudo contribuam para a maior efetividade de estratégias de combate e de prevenção ao uso de substâncias psicoativas por adolescentes.

## INTRODUÇÃO

A adolescência é a etapa da vida compreendida entre a infância e a fase adulta, marcada por um processo complexo de crescimento e de adaptação às novas estruturas físicas, psicológicas e ambientais.^1^ Ao se somarem a esse processo fatores socioeconômicos, como a baixa renda familiar, fatores biológicos e genéticos, relacionados ao desenvolvimento neurológico, além de disfunções familiares e certas exposições ambientais, como o abuso de substâncias e situações de violência, cria-se um ambiente propício para o surgimento de transtornos mentais nessa população.^2^ ^),(^ ^3^ Segundo dados da Organização Mundial da Saúde, um em cada sete jovens de 10 a 19 anos apresentam algum tipo de transtorno mental.^4^

O Estatuto da Criança e do Adolescente, lei que dispõe sobre a proteção integral à criança e ao adolescente, apresenta os cuidados em saúde mental como uma das suas diretrizes básicas, incluindo os relacionados ao uso de álcool e outras substâncias psicoativas.^5^ Apesar desse amparo normativo, o II Relatório Brasileiro sobre Drogas evidenciou o início cada vez mais prematuro do uso dessas substâncias, o que corrobora a ocorrência de transtornos mentais e comportamentais associados a esse uso, além de possíveis quadros agudos que demandam atendimento hospitalar.^6^

O levantamento nacional acerca do uso de drogas realizado em 2017, que incluiu 138 estratos demográficos, evidenciou que aproximadamente 7 milhões (34,3%) de pessoas menores de 18 anos reportaram ter consumido álcool pelo menos uma vez na vida e 22,2% haver consumido nos últimos 12 meses.^7^ Ainda segundo este levantamento, cerca de 1,3 milhão de adolescentes de 12 a 17 anos já haviam consumido cigarros industrializados na vida, e, para aproximadamente 15 milhões de indivíduos (12 a 65 anos) que referiram ter usado alguma substância ilícita durante a vida, a mediana da idade de primeiro consumo foi de 16,6 anos.^7^

Nessa perspectiva, quanto mais precoce a experimentação dessas substâncias, maiores serão os riscos da ocorrência de danos, sejam eles de curto, médio ou longo prazo, diretos ou indiretos. Dentre as possíveis consequências, observam-se: danos à saúde física em diferentes órgãos, conflitos familiares, prejuízo no desenvolvimento, dificuldades escolares e de relacionamento interpessoal, além do risco de envolvimento com criminalidade e violência, em decorrência da circulação em diferentes ambientes, sem supervisão de responsáveis.^8^ ^)^

O uso de substâncias psicoativas e as implicações deste uso para os adolescentes têm sido alvo de diversos estudos brasileiros.^9^ ^),(^ ^10^ ^),(^ ^11^ Contudo, há escassez de estudos sobre as hospitalizações por transtornos mentais devido ao uso de substâncias psicoativas nesse público específico que englobem dados amplos do país, considerando as cinco regiões.Desenvolver estudos com essa abordagem é importante para o desenvolvimento de políticas de saúde mental mais integradas e eficazes.

Considerando a importância da temática dos transtornos mentais relativos ao uso de substâncias psicoativas na adolescência e a escassez de estudos com esse enfoque, este trabalho teve como objetivo analisar o perfil epidemiológico e a tendência das internações hospitalares por transtornos mentais e comportamentais devido ao uso de álcool e de outras substâncias psicoativas em adolescentes brasileiros, no período de 2017 a 2022.

## MÉTODOS

*Desenho do estudo*

Trata-se de estudo de série temporal, com análise das internações hospitalares por transtornos mentais e comportamentais devido ao uso de álcool e de outras substâncias psicoativas em adolescentes no Brasil, no período de 2017 a 2022, utilizando-se o número de internações registradas por local de internação como unidade de análise.

*Contexto*

De acordo com o último censo divulgado pelo Instituto Brasileiro de Geografia e Estatística (IBGE), o Brasil tinha 203.062.512 habitantes em 2022, dos quais 28.050.903 estavam na faixa etária de 10 a 19 anos. O Brasil é composto de 5.570 municípios, distribuídos em 27 Unidades da Federação e cinco macrorregiões nacionais, em ordem decrescente de população: Sudeste (41,8%), Nordeste (26,9%), Sul (14,7%), Norte (8,5%) e Centro-Oeste (8,0%).^12^ Segundo o Cadastro Nacional de Estabelecimentos Saúde, em 2023, o Brasil apresentava 32.097 leitos destinados a psiquiatria e saúde mental, sendo 17.280 leitos disponíveis para internação pelo Sistema Único de Saúde (SUS).^13^

Os dados acerca das hospitalizações são oriundos do Sistema de Informações Hospitalares (SIH) do SUS, gerido pelo Ministério da Saúde, pela Secretaria de Assistência à Saúde, em conjunto com as Secretarias Estaduais de Saúde e as Secretarias Municipais de Saúde. Esses dados foram extraídos do sítio eletrônico do Departamento de Informática do Sistema Único de Saúde (DATASUS) mediante consulta em junho de 2023.^14^

*Participantes*

Foram selecionados os registros de internação com diagnósticos codificados como F10 (Transtornos mentais e comportamentais devido ao uso de álcool) e F19 (Transtornos mentais e comportamentais devido ao uso de outras substâncias psicoativas), conforme Capítulo V da 10ª Revisão da Classificação Estatística Internacional de Doenças e Problemas relacionados à Saúde (CID-10).^15^

*Variáveis*

As variáveis utilizadas para análise das internações hospitalares por transtornos mentais e comportamentais devido ao uso de álcool e outras substâncias psicoativas em adolescentes foram: ano de atendimento (2017 a 2022), macrorregião de residência (Norte, Nordeste, Sudeste, Sul e Centro-Oeste), sexo (masculino ou feminino), raça/cor da pele (branca, preta, parda, amarela e indígena), faixa etária (10 a 14 e 15 a 19 anos), média de permanência hospitalar (dias) e caráter do atendimento (eletivo ou urgência). 

*Coleta de dados*

Foram utilizados os dados do SIH/SUS e as projeções da população adolescente residente (10 a 19 anos), disponibilizada pelo IBGE para o período de 2017 a 2022.^14^

*Análise de dados*

Para o cálculo da taxa de internação, utilizou-se como numerador o número de internações hospitalares em adolescentes por transtornos mentais e comportamentais devido ao uso de álcool e outras substâncias psicoativas disponibilizado pelo SUS, e no denominador, a projeção do IBGE da população de adolescentes residentes no mesmo local e período considerado, multiplicada por 100 mil. 

A tendência temporal foi analisada utilizando-se o método de Prais-Winsten para regressão linear generalizada. Nesse método, os anos da série histórica (de 2017 a 2022) foram considerados como variáveis independentes (X), enquanto os logaritmos das taxas de internação por região de residência, sexo e faixa etária (10 a 14 e 15 a 19 anos) foram considerados como variáveis dependentes (Y). A transformação logarítmica de base 10 aplicada às variáveis dependentes (Y) tem o propósito de reduzir a heterogeneidade da variância dos resíduos na análise de regressão linear, auxiliando na identificação da tendência.^16^ Esse modelo de regressão é apropriado para corrigir a autocorrelação serial em séries temporais, que se refere à dependência de medida ao longo do tempo com seus próprios valores em momentos anteriores. A autocorrelação serial foi avaliada através do teste de Durbin-Watson.^16^

A tendência foi estimada pelo cálculo da variação percentual anual (VPA) = [-1 + 10b1] * 100%. O intervalo de confiança correspondente (IC_95%_) foi obtido utilizando-se a fórmula = [-1 + 10b1mín.] * 100% até [-1 + 10b1máx.] * 100%. As tendências foram interpretadas da seguinte maneira: crescentes quando a variação era positiva, decrescentes quando a variação era negativa, e estáveis quando não havia diferença significativa. Resultados com p-valor <0,05 foram considerados significativos.^16^

As análises dos dados foram realizadas utilizando-se o *software* R versão 4.2.2, considerando-se um nível de confiança de 95%.

*Aspectos éticos*

Por se tratar de dados secundários, disponibilizados pelo DATASUS, o presente estudo não necessitou de apreciação por Comitê de Ética em Pesquisa, em consonância com a Resolução do Conselho Nacional de Saúde CNS nº 466, de 12 de dezembro de 2012.^17^

## RESULTADOS

No período de 2017 a 2022, foram registradas 29.991 internações por transtornos mentais e comportamentais devido ao uso de álcool e outras drogas em adolescentes no Brasil. Observou-se que 72,1% (n = 21.624) das internações ocorreram no sexo masculino e que 86,6 % (n = 25.960) era da faixa etária de 15 a 19 anos. Em relação à raça/cor da pele, a branca foi a mais frequente, representando 44,9% (n = 13.467) das internações no período (Tabela 1).

**Tabela 1:** Características das internações por transtornos mentais e comportamentais devido ao uso de álcool e outras substâncias psicoativas (n = 29.991) registradas no Sistema de Informações Hospitalares do Sistema Único de Saúde, Brasil, 2017-2022

Variáveis	2017	2018	2019	2020	2021	2022	Total	%
Sexo								
Masculino	4.120	4.326	4.190	3.046	2.956	2.986	21.624	72,1
Feminino	1.315	1.513	1.670	1.224	1.281	1.364	8.367	27,9
Faixa etária (anos)								
10-14	679	820	815	586	542	589	4.031	13,4
15-19	4.756	5.019	5.045	3.684	3.695	3.761	25.960	86,6
Raça/cor da pele								
Branca	2.337	2.772	2.665	1.985	1.732	1.976	13.467	44,9
Preta	283	284	354	222	214	275	1.632	5,4
Parda	1.436	1.569	1.694	1.279	1.370	1.444	8.792	29,3
Amarela	61	64	65	47	60	40	337	1,1
Indígena	7	8	10	9	4	2	40	0,1
Sem informação	1.311	1.142	1.072	728	857	613	5.723	19,1

As taxas de internação referentes ao Brasil apresentaram redução de 16,18/100 mil habitantes para 13,72/100 mil habitantes e tendência decrescente entre os anos de 2017 e 2022 (Figura 1 e Tabela 2).

**Figura 1:** Distribuição das taxas de internação por transtornos mentais e comportamentais referentes ao uso de álcool e outras substâncias psicoativas em adolescentes, por região, Brasil, 2017-2022

**Tabela 2:** Taxas anuais e tendência das internações por 100 mil habitantes, por transtornos mentais e comportamentais devido ao uso de álcool e outras substâncias psicoativas em adolescentes (idade entre 15 e 19 anos), segundo região, sexo e faixa etária, Brasil, 2017-2022

Variáveis	2017	2018	2019	2020	2021	2022	Coeficiente	p-valor	Variação (%)	IC_95%​_^a^	Tendência^b^
Região											
Norte	​​4,27​	​​4,55​	​​3,08​	​​2,29​	​​2,68​	​​2,82​	​​-0,05	​​0,080​	​​-4,7	​​-8,42;-0,77	​​ Estável
Nordeste	​​7,22​	​​7,86​	​​7,34​	​​5,86​	​​6,96​	​​6,65​	​​-0,01	​​0,152​	​​-1,4​	​​-2,92;0,16​	Estável
Sudeste	​​12,43​	​​13,29​	​​13,88​	​​10,30​	​​10,38​	​​12,38​	​​-0,02	​​0,212​	​​-1,6​	​​-3,74;0,53​	Estável
Sul	​​58,43​	​​65,70​	​​66,34​	​​48,77​	​​46,42​	​​45,55​	​​-0,03	​​0,039​	​​-3,3​	​​-5,37;-1,16	Decrescente
Centro-Oeste	​​14,79​	​​13,48​	​​17,12​	​​10,86​	​​10,76​	​​10,57​	​​-0,04	​​0,030​	​​-3,6​	​​-5,75;-1,49	Decrescente
Brasil	​​16,18​	​​17,53​	​​17,77​	​​13,10​	​​13,18​	​​13,72​	​​-0,03	​​0,049​	​​-2,7​	​​-4,47;-0,80	Decrescente
Sexo											
Masculino	​​24,07​	​​25,48	​​24,92​	​​18,32​	​​18,02​	​​18,45​	​​-0,03	​​0,018	​​-3,5​	​​-5,20;-1,72	Decrescente
Feminino	​​7,98​	​​9,26​	​​10,33​	​​7,66​	​​8,13​	​​8,78​	​​-0,01	​​0,683​	​​-0,5​	​​-2,65;1,72​	Estável
Faixa etária (anos)											
10 a 14	​​4,13​	​​5,06​	​​5,10​	​​3,72​	​​3,49​	​​3,85​	​​-0,02	​​0,163	​​-2,4​	​​-5,02;0,36​	Estável
15 a 19	​​27,73​	​​29,37​	​​29,70​	​​21,87​	​​22,23​	​​22,93​	​​-0,03	​​0,035​	​​-2,8​	​​-4,49;-1,06	Decrescente

a) IC_95%_: Intervalo de confiança de 95%; b) Tendência decrescente, quando p-valor < 0,05 e o coeficiente de regressão for negativo.

As taxas de internação mais elevadas ocorreram na região Sul, variando de 58,4/100 mil hab. para 45,6/100 mil hab. no período estudado. As regiões Sul (-3,3%; IC_95%_ -5,37;-1,16) e Centro-Oeste (-3,6%; IC_95%_ -5,75;-1,49) apresentaram tendências decrescentes das taxas de internação, enquanto as demais regiões mantiveram estáveis (Tabela 2).

As taxas de internação do sexo masculino apresentaram tendência decrescente (-3,5%; IC_95%_ -5,20;-1,72), variando de 24,07/100 mil hab. para 18,45/100 mil hab., enquanto as taxas do sexo feminino foram estáveis (Tabela 2 e Figura 2). Em relação às faixas etárias analisadas, observou-se tendência decrescente das taxas de internação na faixa de 15 a 19 anos (-2,8%; IC_95%_ -4,49;-1,06), variando de 27,73/100 mil hab. para 22,93/100 mil hab. (Tabela 2). 

**Figura 2:** Distribuição das taxas de internação por transtornos mentais e comportamentais referentes ao uso de álcool e outras substâncias psicoativas em adolescentes, por sexo, Brasil, 2017-2022

O tempo médio de permanência hospitalar, referente às autorizações de internação hospitalar aprovadas no período em análise, foi de 16,4 dias em 2017 e de 15,8 dias em 2022. A região Nordeste apresentou a maior média (19,4 dias), seguida da região Sul (18,5 dias), região Sudeste (14,4 dias), região Centro-Oeste (8,5 dias) e região Norte (6,2 dias). Já em relação ao caráter de atendimento, observou-se que a maioria das internações ocorreu em caráter de urgência, representando 88,3% (26.483) do total.

## DISCUSSÃO

No Brasil, de 2017 a 2022, as taxas de internação por transtornos mentais e comportamentais devido ao uso de álcool e outras substâncias psicoativas em adolescentes registraram tendência decrescente, com maior declínio no sexo masculino, na faixa etária de 15 a 19 anos e nas regiões Sul e Centro-Oeste. Destaca-se que as demais variáveis utilizadas para a análise das taxas de internação apresentaram tendência estável.

Na análise do período em questão, a região Sul apresentou número mais expressivo de internações. De acordo com o *Estudo dos Riscos Cardiovasculares em Adolescentes* com estudantes de 12 a 17 anos, o qual fornece apoio substancial aos dados encontrados no presente estudo, o consumo de álcool entre adolescentes também foi maior na região Sul.^18^ ^)^ Uma análise de série temporal realizada no Paraná evidenciou que a principal causa de internações em adolescentes foram transtornos mentais e comportamentais causados pelo uso de substâncias psicoativas, representando 64,35% das internações entre 2012 e 2015.^10^ Uma possível explicação para esse fenômeno é que a percepção sobre o uso de substâncias psicoativas diverge conforme regiões. Segundo um estudo sobre o comportamento de adolescentes em relação ao consumo de álcool, no Norte há mais relatos de medo e insegurança da dependência química, enquanto no Sul é comum considerar o álcool menos prejudicial à saúde. Destaca-se, nessa região, uma cultura em torno do consumo de vinho, influenciada pelas tradições alemã e italiana, moldando a maneira como essa bebida é apreciada e cultuada.^19^

O sexo masculino concentrou a maior parte das internações. Um estudo que abordou as internações por transtornos mentais e comportamentais devido ao uso de álcool em jovens brasileiros, entre 2010 e 2020, também constatou taxa de internações mais elevada entre os jovens do sexo masculino, confirmando, assim, os achados observados neste estudo.^20^ Essa disparidade entre os sexos pode ser atribuída a várias influências, como, por exemplo, a cultura nacional, marcada por persistente rotulação do papel de gênero, e os meios social e midiático, que influenciam o acesso e a aceitabilidade do consumo dessas substâncias pelo sexo masculino.^21^ Similarmente, de acordo com os resultados encontrados no *II Relatório Brasileiro sobre Drogas*, os homens apresentaram taxas de internação mais elevadas em todas as faixas etárias, sendo este um padrão consistente em todos os estados do Brasil.^6^ Isso sugere que não ocorreram alterações significativas na distribuição das internações segundo sexo relacionadas à utilização de substâncias psicoativas ao longo desse período do estudo. 

Analisando-se os resultados segundo faixa etária, nota-se que as internações obtiveram taxas maiores no intervalo de 15 a 19 anos. É possível que esse fato esteja relacionado com a licitude do álcool no país e ao amplo acesso à substância, com difícil fiscalização de consumo, apesar das restrições legais para menores de idade. Além disso, é possível haver correlação com a influência significativa das campanhas publicitárias que promovem o uso de álcool e com a pressão social exercida por grupos que incentivam o consumo dessa substância.^11^ Também os contextos, sejam familiares, educacionais ou sociais que cada faixa etária frequenta, desempenham papel significativo no consumo e nas internações. Por exemplo, jovens a partir dos 18 anos têm fácil acesso a bares e boates, o que pode aumentar o consumo. Esses jovens também estão expostos a ambientes universitários, nos quais os colegas frequentemente incentivam o uso de álcool e outras substâncias.^22^

O tempo de instalação da dependência química é variável, uma vez que sofre influência de fatores genéticos, ambientais, psicossociais e neurobiológicos. A exposição a drogas durante a adolescência pode aumentar a vulnerabilidade ao desenvolvimento da dependência química, devido às mudanças cerebrais significativas que ocorrem nessa faixa etária, como o desenvolvimento de circuitos de recompensa e de regulação emocional e a plasticidade sináptica. Esta, uma vez alterada, pode causar adaptações duradouras nos circuitos cerebrais, aumentando o risco da dependência, o que reforça o impacto da faixa etária na iniciação e manutenção do uso de substâncias psicoativas.^23^

A redução das taxas de internações entre 2017 e 2022 pode ser atribuída a uma série de fatores, como a restrição do acesso aos serviços de saúde e a implementação de políticas de distanciamento social durante a pandemia da covid-19. Segundo a Organização Mundial da Saúde, aproximadamente 35% dos serviços de emergência relacionados à saúde mental foram interrompidos.^24^Em um hospital universitário do Rio Grande do Sul, no período de outubro de 2019 a outubro de 2020, houve redução de 30 para 20 leitos por 60 dias como medida preventiva.^25^ Outro estudo, realizado nos Estados Unidos em 2020, constatou uma redução geral do uso de substâncias psicoativas por adolescentes durante a pandemia.^26^ ^)^ Esses achados demonstram o impacto da pandemia nas hospitalizações por transtornos mentais e comportamentais, as quais declinavam em 2020 em comparação a 2019, seguido por um aumento em 2021.^27^

A maioria das hospitalizações ocorreu em caráter de urgência, um dado respaldado por um estudo conduzido na região Norte do Brasil, entre 2017 e 2021, na qual 97% das internações foram na urgência.^27^ A intoxicação aguda foi descrita como a apresentação clínica mais frequente do abuso de substâncias entre adolescentes, sendo manifestada com agitação psicomotora, agressividade, psicose aguda e, nos casos mais graves, confusão mental, e coma.^28^ Contudo, não é possível determinar se as internações se deram devido à própria intoxicação ou se devido a complicações secundárias a esta, uma vez que a ficha de notificação não fornece espaço para tal distinção.

Em um estudo com menores de 18 anos usuários de substâncias no serviço de emergência psiquiátrica em São Paulo, entre 2000 e 2011, destacou-se que houve aumento da frequência de utilização desses serviços, que estão relacionados à crescente prevalência de problemas de saúde mental nessa faixa etária, à dificuldade de acesso aos serviços de saúde comunitários e ao estigma associado aos transtornos mentais nos demais estabelecimentos de saúde.^29^ Frequentemente, a emergência psiquiátrica é a primeira e até mesmo a única fonte de cuidado para a saúde infanto-juvenil, sendo fundamental o reconhecimento da importância desses serviços para o desenvolvimento de protocolos de intervenção eficazes, com diretrizes claras de ação, encaminhamento e retorno, bem como para o desenvolvimento de condutas que abarquem os fatores psicossociais que permeiam essa demanda. 

O presente estudo apresenta algumas limitações inerentes ao seu desenho. As informações agregadas por região geográfica ou local podem obscurecer as nuances individuais e os fatores de confusão. Problemas relacionados ao preenchimento incorreto ou incompleto das informações nos registros hospitalares também podem estar presentes. Erros de codificação de diagnósticos e procedimentos, bem como falta de detalhamento sobre as condições clínicas dos pacientes, podem comprometer a qualidade dos dados analisados, dificultando a avaliação precisa dos resultados. 

Outra limitação diz respeito às questões socioeconômicas, pois o estudo pode não capturar completamente a diversidade da população que necessita de cuidados médicos. A exclusão daqueles que não procuraram atendimento hospitalar ou optaram por assistência médica privada introduz viés, resultando em subestimação da prevalência real dos transtornos mentais e comportamentais relacionados ao uso de substâncias psicoativas. Portanto, é fundamental reconhecer essas limitações na interpretação dos resultados e considerar abordagens complementares, como estudos de coorte ou pesquisas qualitativas, para se obter compreensão mais abrangente e precisa dessas questões de saúde pública, levando em conta as disparidades socioeconômicas na população.

Constata-se que, no período analisado, houve tendência decrescente das hospitalizações por transtornos mentais e comportamentais devido ao uso de substâncias psicoativas entre adolescentes, com disparidades entre as regiões e maior prevalência das internações entre jovens do sexo masculino, da raça/cor da pele branca e da faixa etária de 15 a 19 anos. Diante desses resultados, é crucial salientar a importância deste estudo, uma vez que fornece informações essenciais para o planejamento e direcionamento das políticas públicas e práticas em saúde mental voltadas ao público adolescente. Contudo, ressalta-se a necessidade de pesquisas adicionais que aprofundem a discussão e proporcionem novas contribuições, como estudos longitudinais ou intervenções controladas, além de pesquisas que explorem informações em nível individual, como fatores socioeconômicos, histórico familiar e acesso a programas de prevenção e intervenção, o que permitiria identificar grupos de maior vulnerabilidade e orientar a formulação de intervenções mais direcionadas.
